# Association Between Food Habits with Mental Health and Executive Function in Chilean Children and Adolescents

**DOI:** 10.3390/children12030268

**Published:** 2025-02-22

**Authors:** Felipe Caamaño-Navarrete, Indya del-Cuerpo, Carlos Arriagada-Hernández, Mauricio Cresp-Barria, Claudio Hernández-Mosqueira, Guido Contreras-Díaz, Pablo Valdés-Badilla, Daniel Jerez-Mayorga, Pedro Delgado-Floody

**Affiliations:** 1 Physical Education Career, Universidad Autónoma de Chile, Temuco 4780000, Chile; marfel77@gmail.com (F.C.-N.); carlos.arriagada@uautonoma.cl (C.A.-H.); 2 Collaborative Research Group for School Development (GICDE), Temuco 4780000, Chile; 3 Department of Physical Education and Sport, Faculty of Sports Sciences, University of Granada, 18012 Granada, Spain; delcuerpo@ugr.es (I.d.-C.);djerezmayorga@ugr.es (D.J.-M.); 4 Departamento de Educación e Innovación, Universidad Católica de Temuco, Temuco 4780000, Chile; mcresp@uct.cl; 5 Departamento de Ciencias de la Educación, Universidad del Bio-Bio, Concepción 4051381, Chile; chernandez@ubiobio.cl; 6 Escuela de Kinesiología, Facultad de Odontología y Ciencias de la Rehabilitación, Universidad San Sebastián, Lago Panguipulli 1390, Puerto Montt 5501842, Chile; guido.contreras@uss.cl; 7 Department of Physical Activity Sciences, Faculty of Education Sciences, Universidad Católica del Maule, Talca 3530000, Chile; pvaldes@ucm.cl; 8 Sports Coach Career, School of Education, Universidad Viña del Mar, Viña del Mar 2580022, Chile; 9 Exercise and Rehabilitation Sciences Institute, School of Physical Therapy, Faculty of Rehabilitation Sciences, Universidad Andres Bello, Santiago 7591538, Chile; 10 Department of Physical Education, Sport and Recreation, Universidad de La Frontera, Temuco 4811230, Chile

**Keywords:** children, foods habits, depression, anxiety, stress, cognitive flexibility

## Abstract

Objective: To determine the association between foods habits with mental health (i.e., anxiety, depression, and stress) and executive function (i.e., attention, inhibition, working memory, and cognitive flexibility) in Chilean children and adolescents. Methods: A cross-sectional study with 498 children and adolescents (52.6% female) aged 10–17 years participated. The Krece Plus questionnaire (Food habits), Depression Anxiety Stress Scales (DASS-21, metal health), and the CogniFit (executive functions) test were used to assess the study variables. Results: The poor and moderate food habits groups reported higher prevalence of extremely severe anxiety (poor, 40.8%; moderate, 41.4%; good, 21.6%; *p* = 0.013) and extremely severe depression (poor, 20.4%; moderate, 21.3%; good, 5.7%; *p* < 0.001). The food habits were linked inversely to anxiety (β −0.07, 95%CI −0.11 to −0.03, *p* = 0.001), depression (β −0.08, 95%CI −0.12 to −0.04, *p* < 0.001), stress (β −0.07, 95%CI −0.11 to −0.02, *p* = 0.004), and total score of negative mental health (β −0.03, 95%CI −0.04 to −0.01, *p* < 0.001). Conclusions: The food habits were inversely associated with negative metal health in Chilean children and adolescents, where the good food habits group reported better mental health in all dimensions.

## 1. Introduction

Childhood and adolescence are crucial life stages characterized by significant physiological and psychological changes [1], where humans develop new cognitive skills (including abstract thinking capacities), a clearer sense of personal and sexual identity, and a degree of emotional and personal growth. Hence, adolescence is increasingly recognized as a life period that poses specific challenges for treating disease and promoting health. Mental health and cognitive development, particularly executive functions (EFs) [2], are critical determinants of later life outcomes. Mental health is widely recognized as a key component of well-being [3], while EFs play a central role in learning, decision-making, and academic success [4]. They allow you to solve problems and manage emotions; research suggests that strong executive functioning skills make a difference in your mental and physical health and quality of life. 

These functions are essential not only for mental health but also for academic achievement and overall [5]. Good mental health fosters the development of life skills, acts as a protective factor against stress, and contributes positively to relationships with peers and family [6]. Additionally, mental health is strongly linked to both social and psychological functioning [7]. However, mental health issues during the school years (i.e., childhood and adolescence) represent a significant global public health challenge [8]. Consequently, promoting positive mental health among adolescents has become a priority in health and educational policies across Latin American countries [9]. Moreover, EFs have been identified as strong predictors of academic performance [6]. Among the core EFs, inhibitory control, working memory, and cognitive flexibility are the primary areas of focus in scientific research [10]. Studies suggest that emphasizing EFs such as attention, inhibition, working memory, and cognitive flexibility may facilitate targeted interventions that enhance self-regulation skills, ultimately fostering resilience.

Children’s food habits are crucial determinants of mental health. Evidence has shown that fruit and vegetable consumption is associated with better mental health in adolescents, whereas lower frequencies of fruit intake and breakfast consumption have been identified as significant risk factors for mental health issues, particularly among females [11]. Accumulating scientific evidence highlights the role of food habits in mental health promotion and prevention. Healthy dietary patterns have been inversely associated with depression incidence [12]. A nationally representative study examining the associations between adolescent eating habits and mental health—while accounting for age, sex, and socioeconomic factors—across five Nordic countries (Denmark, Finland, Iceland, Norway, and Sweden) found that healthier food habits, such as regular family meals and breakfast consumption, were linked to improved mental health outcomes [13]. Furthermore, adherence to the Mediterranean diet has been associated with positive health outcomes, including a reduced incidence of mental health disorders. Thus, the Mediterranean diet may serve as a protective factor for mental health among children and adolescents [14]. Additionally, evidence supports a strong association between healthier food habits and improved psychosocial well-being, with high-quality dietary patterns being significantly linked to lower levels of depressive symptoms [15].

Healthy food habits have been linked to better executive functions (EFs). In contrast, unhealthy dietary patterns, such as frequent consumption of processed snacks, have shown an inverse association with EFs in children and adolescents [16]. Furthermore, life satisfaction in children and adolescents has been positively associated with healthier food habits, including a higher frequency of breakfast consumption among individuals aged 10 to 17 years across 42 different countries [17]. Additionally, in Chilean children and adolescents, greater adherence to the Mediterranean diet (i.e., healthy eating habits) has been linked to improved cognitive and academic performance [18].

Given these findings, it is crucial to examine food habits in relation to the Mediterranean diet and their effects on mental health and EFs in children and adolescents. However, to the best of our knowledge, limited information exists regarding the association between food habits, mental health, and EFs in Chilean children and adolescents. Therefore, this study aimed to determine the relationship between food habits and mental health (i.e., anxiety, depression, and stress) as well as executive function (i.e., attention, inhibition, working memory, and cognitive flexibility) in this population. We hypothesize that better food habits are related to mental health (i.e., anxiety, depression, and stress) and executive function (i.e., attention, inhibition, working memory, and cognitive flexibility) in Chilean children and adolescents.

## 2. Materials and Methods

### 2.1. Participants

In this cross-sectional study, 498 Chilean students aged 10 to 17 years from various schools in the city of Temuco, Chile, participated actively. The sex distribution was nearly equal, with 249 males and 262 females. A total of 53 students were excluded during the selection process. Reasons for exclusion included female students who did not meet the inclusion criteria or had personal circumstances preventing participation (*n* = 30), and male students who similarly did not meet the inclusion criteria or faced other barriers to participation (*n* = 23). It is important to note that the sample selection was intentional and non-probabilistic, meaning that participants were chosen deliberately based on predefined research objectives and criteria established by the researchers.

The inclusion criteria for this study were rigorous and designed to ensure the integrity of the research. These criteria included (i) obtaining informed consent from participants and their parents or guardians, thereby ensuring their understanding and voluntary participation in the study, and (ii) being a student, to clearly define the target population. Similarly, the exclusion criteria were detailed to ensure the suitability of participants for the assessments. These included (i) any medical contraindication that could impair normal performance in the assessments, thus ensuring that the results accurately reflected the capabilities of the student population under study, and (ii) absence during the assessments or failure to provide informed consent, thereby maintaining the validity and ethical standards of data collection. This study strictly adhered to the ethical principles outlined in the Declaration of Helsinki (2013), ensuring the protection of participants’ rights in scientific research. Additionally, it received approval from the Ethics Committee of Universidad Autónoma de Chile, Chile (ACTA; No. CEC 11-23), affirming the study’s methodological validity and ethical integrity. Student participation was contingent upon obtaining their signed consent, along with the informed consent of their respective parents or guardians.

### 2.2. Main Outcomes

#### 2.2.1. Food Habits

The Krece Plus questionnaire was used to assess food habits and determine dietary quality based on adherence to the Mediterranean Diet [19]. This test is a valuable tool for predicting and preventing nutritional imbalances in children and adolescents. It allows for the rapid and effective detection of nutritional and physical risks that may contribute to obesity. The instrument consists of 15 dichotomous questions (answered with “yes” or “no”), each scored as either +1 or −1 according to established guidelines. The total score is classified based on previous recommendations as follows: (i) 8 to 12: good food habits; (ii) 4 to 7: moderate food habits (indicating a need for dietary improvement); and (iii) 0 to 3: poor food habits (indicating very low diet quality) [19]. The Krece Plus instrument has been previously validated and applied to Chilean students [20]. The questionnaire items used are presented in Table 1.

#### 2.2.2. Mental Health

The abbreviated version of the Depression Anxiety Stress Scales (DASS-21) was used to assess mental health problems [21]. This three-dimensional self-report scale is designed to evaluate the presence and severity of emotional states or symptoms related to depression, anxiety, and stress [22,23,24]. The questionnaire consists of 21 items divided into three subscales: depression (assessing dysphoria, hopelessness, devaluation of life, self-hatred, lack of interest, and anhedonia), anxiety (evaluating physiological arousal, situational anxiety, and general anxiety), and stress (measuring irritability, feelings of being overwhelmed, difficulty relaxing, and excessive thinking). Each item is rated on a scale from 0 to 3 based on symptom intensity over the past week, ranging from “It does not describe anything that happened to me or how I felt during the week” (0) to “Yes, this happened to me a lot, or most of the time” (3). The total score ranges from 0 to 21. This instrument is advantageous due to its brevity, ease of administration and interpretation, and its effectiveness as a self-report tool [25]. The DASS-21 has been previously validated and applied to Chilean student populations [26] demonstrating adequate psychometric properties [25].

#### 2.2.3. Executive Function

To assess executive functions (EFs), including inhibition, working memory, cognitive flexibility, and attention, the CogniFit neurocognitive assessment battery (San Francisco, CA, USA) was utilized [27]. This 40 min evaluation provides both an overall cognitive score and specific scores for individual EFs. The CogniFit battery has demonstrated good reliability and has been successfully implemented with school-aged children [28].

The neuropsychological test was administered online and required approximately 30 to 40 min to complete. Upon completion, a comprehensive results report was automatically generated, outlining the user’s neurocognitive profile. Previous studies have confirmed that this cognitive profile exhibits high reliability, consistency, and stability [29].

#### 2.2.4. Procedure

The research team responsible for evaluating the adolescent participants was trained in the study protocols and assessment procedures prior to data collection. Assessments related to mental health, lifestyle, and active commuting were conducted during a morning class session in a laboratory setting with computers provided by the schools. Participants completed the questionnaires and evaluations individually under the supervision of researchers, who were available to address any questions or concerns. Data collection took place between April and November 2023, aligning with the academic calendar of the adolescents.

### 2.3. Statistical Analysis

Statistical analyses were performed using SPSS^®^ v23.0 software (SPSS Inc., Chicago, IL, USA). The normality of the data was assessed using the Kolmogorov–Smirnov test. Continuous variables are presented as mean and standard deviation (SD), while categorical variables are expressed as frequency and percentage using the Chi-square test. Differences between sexes were analyzed using the Student’s *t*-test, while group comparisons were conducted using ANOVA with the appropriate post hoc analysis. To examine the association between mental health, executive functions (EFs), and food habits, a simple linear regression analysis was performed, with results reported as beta coefficients (β) and their 95% confidence intervals (CIs). All analyses were adjusted for sex and age. A *p*-value < 0.05 was considered statistically significant.

## 3. Results

Table 2 presents the characteristics of the study sample and a comparison by sex. Males reported better mental health outcomes than females in anxiety (Male: 6.31 ± 5.09 vs. Female: 9.42 ± 5.80, *p* < 0.001), depression (Male: 6.78 ± 4.99 vs. Female: 9.33 ± 5.78, *p* < 0.001), stress (Male: 8.37 ± 4.93 vs. Female: 10.98 ± 5.24, *p* < 0.001), and overall mental health (total score: Male: 21.45 ± 13.48 vs. Female: 29.73 ± 15.60, *p* < 0.001). Regarding executive functions (EFs), significant differences were observed in attention (Male: 445.01 ± 148.99 vs. Female: 399.14 ± 154.06, *p* = 0.001) and cognitive flexibility (Male: 392.96 ± 248.50 vs. Female: 313.05 ± 236.84, *p* < 0.001).

Table 3 presents the comparison of mental health and executive functions according to food habits categories (poor, moderate, and good). Significant differences were observed between the poor–moderate and good food habits groups in anxiety (*p* < 0.001), depression (*p* < 0.001), stress (*p* = 0.014), and DASS total score (*p* < 0.001). However, no significant differences were found in executive functions.

Table 4 presents the frequency of mental health problems, including anxiety, depression, stress, and total mental health score, according to food habits categories. The poor and moderate food habits groups exhibited a higher prevalence of extremely severe anxiety compared to the good food habits group (poor: 40.8%; moderate: 41.4%; good: 21.6%, *p* = 0.013). Similarly, the poor and moderate food habits groups had a higher prevalence of extremely severe depression (poor: 20.4%; moderate: 21.3%; good: 5.7%, *p* < 0.001). However, no significant differences were observed between groups regarding stress levels.

Table 5 presents the association between food habits, mental health, and executive function. Food habits were inversely associated with anxiety (β = −0.07, 95% CI: −0.11 to −0.03, *p* = 0.001), depression (β = −0.08, 95% CI: −0.12 to −0.04, *p* < 0.001), stress (β = −0.07, 95% CI: −0.11 to −0.02, *p* = 0.004), and the total score of negative mental health (β = −0.03, 95% CI: −0.04 to −0.01, *p* < 0.001). However, no significant associations were found between food habits and executive functions.

## 4. Discussion

The main aim of this study was to examine the association between food habits and mental health (i.e., anxiety, depression, and stress) as well as executive function (i.e., attention, inhibition, working memory, and cognitive flexibility) in Chilean children and adolescents. The key findings indicated that poor and moderate food habits were associated with a higher prevalence of extreme anxiety and depression compared to good food habits. However, no significant differences were observed in stress levels across the different food habit categories. Furthermore, no significant associations were found between food habits and executive functions.

Poor and moderate food habits were significantly associated with a higher prevalence of extreme anxiety in children and adolescents, as demonstrated by the study findings. This aligns with prior research, such as a study on dietary patterns among Chinese adolescents, which found that unhealthy food habits, particularly those high in snacks and animal-based foods, were linked to higher odds of anxiety symptoms [30]. Similarly, the CASPIAN-V study reported that skipping breakfast and consuming high amounts of sugary snacks and soft drinks were significantly associated with anxiety and other mental health challenges in children and adolescents [31]. Other studies have shown that adherence to healthier dietary patterns, including a high intake of fruits and vegetables, is associated with lower anxiety levels [32]. These findings highlight the potential impact of unhealthy eating behaviors on mental well-being and emphasize the need to promote healthier food habits as a strategy to mitigate rising anxiety levels among children and adolescents.

A significant association was also found between poor and moderate food habits and a higher prevalence of extreme depression in children and adolescents, according to the findings of this study. These results are supported by previous studies indicating that unhealthy dietary patterns are linked to a greater risk of depression in young people [33,34]. For instance, a study on dietary patterns and mental health in adolescents demonstrated that adherence to unhealthy diets, such as those high in processed foods and snacks, was associated with increased depressive symptoms [35]. Additionally, the CASPIAN-IV study, mentioned earlier, also highlighted that frequent consumption of junk food, such as sweets and fast foods, significantly increased the likelihood of experiencing psychiatric distress, including depression [36]. Additionally, the systematic review published by O’Neil et al. [37] demonstrated a decade ago that adherence to healthy dietary patterns reduces the risk of depressive symptoms, possibly due to the anti-inflammatory and neuroprotective properties of certain nutrients such as omega-3 fatty acids, folate, and antioxidants. Biologically, these nutrients may regulate brain function by modulating neurotransmitter activity, reducing oxidative stress, and promoting neuroplasticity [38].

In our study, no significant differences were found in stress levels based on food habits among Chilean children and adolescents. These results differ from recent studies that have documented associations between dietary habits and perceived stress. For instance, the study conducted by Park et al. [39] on Korean adolescents demonstrated that frequent consumption of unhealthy foods, such as fast food and soft drinks, is associated with higher stress levels, while a higher intake of fruits, vegetables, and milk correlates with better perceptions of general and mental health. Similarly, the study by Michels et al. [40] highlighted that stress can lead to emotional eating patterns, which promote the consumption of foods high in fats and sugars, exacerbating mental health issues. The lack of association could be explained by the limited sample size and cultural differences in how stress is perceived in this specific population. Additionally, contextual factors, such as access to healthy foods and family influence, might have mitigated the impact of stress on dietary habits. Lastly, the cross-sectional design of this study may hinder the ability to capture causal relationships, underscoring the need for longitudinal research.

The present study showed an inverse relationship between food habits and mental health problems according to the DASS-21 scale, where better food habits were associated with a lower prevalence of anxiety, depression, and stress. This finding is supported by recent research such as de Caamaño-Navarrete et al. [15] in which it was observed that a healthy diet was significantly associated with fewer symptoms of anxiety and depression in children and adolescents. Additionally, a study carried out by Geraets and Heinz [41] claimed that adolescents who followed a healthy eating pattern experienced better mental health compared to those with less balanced diets. Furthermore, evidence indicates that healthy diets, rich in fruits, vegetables, and essential nutrients, promote a healthier emotional balance thanks to the anti-inflammatory and neuroprotective effects of nutrients such as omega-3 fatty acids, B complex vitamins, and antioxidants, which reduce oxidative stress and improve brain function [42].

In our study, no significant associations were found between food habits and executive functions, such as attention, inhibition, working memory, and cognitive flexibility. This contrasts with previous research that has shown relationships between a healthy diet and better executive function performance. For example, the study published by Cohen et al. [16] found that healthy dietary patterns, characterized by higher consumption of whole grains, fruits, and vegetables, were positively associated with better executive function, while less healthy foods, such as processed snacks and sugary drinks, showed an inverse association. The HELENA study highlighted that higher dietary quality, measured through healthy diet indices, was related to better attentional control in European adolescents [43]. The lack of association could be explained by methodological factors, such as sample size, sensitivity of the tools, or cultural differences, as well as the cross-sectional design of this study, which limits the ability to establish causal relationships and underscores the need for longitudinal research.

Promoting healthy food habits is essential to improving the mental health of children and adolescents, given the positive impact of a balanced diet on reducing issues like anxiety and depression [14]. These findings highlight the importance of incorporating educational programs and awareness campaigns in schools and communities, as well as developing public policies that facilitate access to healthy foods, especially for vulnerable populations. In the context of Chile, these initiatives could play a key role in preventing mental health problems and promoting overall well-being, serving as a model for implementing similar strategies in other countries.

This study contributes to the field of mental health and food habits research by providing evidence of the inverse relationship between the quality of food habits and mental health issues, such as anxiety and depression, in Chilean children and adolescents. However, no significant associations were identified with executive functions, raising questions about potential underlying mechanisms that remain unexplored. This study has several limitations, including its cross-sectional design, which prevents establishing causal relationships, and the non-probabilistic sampling method, which limits the generalizability of the findings. Additionally, the self-reported assessments of food habits and mental health may be subject to reporting biases. Future studies should include longitudinal designs and more precise assessment tools to investigate causality and consider contextual factors such as family influences, access to healthy foods, and cultural differences.

## 5. Conclusions

Food habits were inversely associated with negative metal health in Chilean children and adolescents, where the good food habits group reported better mental health in all dimensions. Hence, food habit modification could be a cost-effective strategy for the prevention and treatment of mental health problems among children and adolescents.

## Figures and Tables

**Table 1:** Questions related to Mediterranean diet adherence.

1	Skips breakfast
2	Consumes a dairy product for breakfast (yogurt, milk, etc.)
3	Consumes cereals or grains (bread, etc.) for breakfast
4	Has commercially baked goods or pastries for breakfast
5	Takes a fruit or fruit juice every day
6	Consumes a second fruit every day
7	Consumes a dairy product > 1 time/day
8	Consumes fresh or cooked vegetables regularly 1 time/day
9	Consumes fresh or cooked vegetables > 1 time/day
10	Consumes fish regularly (2–3 times/week)
11	Eats at a fast food restaurant ≥ 1 time/week
12	Eats pulses (lentils, beans, more than once a week)
13	Consumes sweets and candy several times every day
14	Consumes pasta or rice almost every day (≥5/week)
15	Uses olive oil at home

**Table 2:** Characteristics of the study sample and comparison by sex.

	Male (*n* = 243)	Female (*n* = 255)	Total (*n* = 498)	*p* Value _(F Value)_
Age (y)	13.63 ± 1.58	13.72 ± 1.71	13.68 ± 1.65	0.561_(0.34)_
Mental health				
Anxiety (score)	6.31 ± 5.09	9.42 ± 5.80	7.90 ± 5.68	*p* < 0.001_(41.37)_
Depression (score)	6.78 ± 4.99	9.33 ± 5.78	8.09 ± 5.55	*p* < 0.001_(28.42)_
Stress (score)	8.37 ± 4.93	10.98 ± 5.24	9.71 ± 5.25	*p* < 0.001_(33.71)_
Mental Health (total score)	21.45 ± 13.48	29.73 ± 15.60	25.69 ± 15.17	*p* < 0.001_(41.01)_
Executive Functions				
Attention (score)	445.01 ± 148.99	399.14 ± 154.06	421.10 ± 153.23	0.001_(11.25)_
Inhibition (score)	286.80 ± 217.76	286.82 ± 235.29	286.81 ± 226.82	0.999_(0.00)_
Working Memory (score)	219.18 ± 218.55	197.33 ± 197.43	207.81 ± 207.90	0.245_(1.36)_
Cognitive Flexibility (score)	392.96 ± 248.50	313.05 ± 236.84	351.38 ± 245.53	*p* < 0.001_(13.33)_

The values are presented as mean ± SD; a *p*-value < 0.05 was considered statistically significant.

**Table 3:** Comparison according to food habits category in mental health and executive functions.

	Poor Food Habits (*n* = 142) A	Moderate Food Habits (*n* = 268) B	Good Food Habits (*n* = 88) C	*p* Value _(F Value)_
Mental health				
Anxiety (score)	8.15 ± 5.56 ^C^	8.41 ± 5.89 ^C^	5.58 ± 4.74 ^A,B^	*p* < 0.001_(8.78)_
Depression (score)	8.58 ± 5.49 ^C^	8.50 ± 5.76 ^C^	5.68 ± 4.31 ^A,B^	*p* < 0.001_(9.86)_
Stress (score)	10.15 ± 5.02 ^C^	9.87 ± 5.41 ^C^	8.19 ± 4.94 ^A,B^	0.014_(4.30)_
Mental Health (total score)	26.88 ± 14.55	26.78 ± 15.83	19.45 ± 12.65 ^A,B^	*p* < 0.001_(8.78)_
Executive Functions				
Attention (score)	421.95 ± 165.68	421.06 ± 148.64	428.12 ± 149.82	0.935_(0.07)_
Inhibition (score)	280.93 ± 230.87	284.17 ± 220.84	310.05 ± 245.61	0.616_(0.48)_
Working Memory (score)	217.42 ± 220.36	200.73 ± 197.70	217.63 ± 217.06	0.679_(0.39)_
Cognitive Flexibility (score)	336.87 ± 254.95	354.97 ± 237.44	377.66 ± 257.83	0.493_(0.71)_

The values are presented as mean ± SD; a *p*-value < 0.05 was considered statistically significant. Superscript letters A, B, or C indicate significant differences between groups: A = poor food habits, B = moderate food habits, and C = good food habits.

**Table 4:** Frequency of mental health problems according to food habits category.

		Category of Food Habits						
		Poor A		Moderate B		Good C		
		*n*	%	*n*	%	*n*	%	*p* Value
Anxiety	Absence	38	26.8% ^C^	69	25.7% ^C^	38	43.2% ^A,B^	0.013
	Mild	6	4.2%	16	6.0%	9	10.2%	
	Moderate	22	15.5%	47	17.5%	13	14.8%	
	Severe	18	12.7%	25	9.3%	9	10.2%	
	Extremely severe	58	40.8% ^C^	111	41.4% ^C^	19	21.6% ^A,B^	
Total		142	100.0%	268	100.0%	88	100.0%	
Depression	Absence	40	28.2% ^C^	83	31.0% ^C^	36	40.9% ^A,B^	<0.001
	Mild	15	10.6%	26	9.7%	23	26.1%	
	Moderate	34	23.9%	62	23.1%	17	19.3%	
	Severe	24	16.9%	40	14.9%	7	8.0%	
	Extremely severe	29	20.4% ^C^	57	21.3% ^C^	5	5.7% ^A,B^	
Total		142	100.0%	268	100.0%	88	100.0%	
Stress	Absence	48	33.8%	92	34.3%	42	47.7%	0.270
	Mild	17	12.0%	32	11.9%	12	13.6%	
	Moderate	23	16.2%	49	18.3%	16	18.2%	
	Severe	36	25.4%	66	24.6%	13	14.8%	
	Extremely severe	18	12.7%	29	10.8%	5	5.7%	
Total		142	100.0%	268	100.0%	88	100.0%	

Data are presented as *n* and proportions (%). A *p*-value of less than 0.05 was considered statistically significant. Superscript letters A, B, or C indicate significant differences between groups: A = poor food habits, B = moderate food habits, and C = good food habits.

**Table 5:** Association between food habits with mental health and executive function.

	β	β (95%CI)	Beta	SE	*p* Value
Mental variables					
Anxiety		−0.07 (−0.11; −0.03)	−0.15	0.02	0.001
		−0.21 ^&^ (−0.39; −0.02) ^&^	−0.10	0.09	0.026
Depression		−0.08 (−0.12; −0.04)	−0.17	0.02	*p* < 0.001
		−0.27 (−0.45; −0.09) ^&^	−0.13	0.09	0.003
Stress		−0.07 (−0.11; −0.02)	−0.13	0.02	0.004
		−0.15 (−0.32; 0.02 ^&^)	−0.08	0.09	0.083
Mental Health		−0.03 (−0.04; −0.01)	−0.16	0.01	*p* < 0.001
		−0.63 (−1.11; −0.14) ^&^	−0.11	0.25	0.012
Executive functions					
Attention		0.00 (0.00; 0.00)	0.05	0.00	0.314
		0.43 (−4.71; 5.57) ^&^	0.01	2.62	0.869
Inhibition		0.00 (0.00; 0.00)	0.05	0.00	0.314
		3.59 (−4.19; 11.36) ^&^	0.04	3.96	0.365
Working Memory		0.00 (0.00; 0.00)	0.02	0.00	0.681
		−0.10 (−7.08; 6.89) ^&^	0.00	3.56	0.978
Cognitive Flexibility		0.00 (0.00; 0.00)	0.08	0.00	0.078
		3.79 (−4.26; 11.83) ^&^	0.04	4.10	0.356

Data shown represent beta and 95% confidence interval β (95%CI). ^&^ represents values adjusted by sex and age.

## Data Availability

The original contributions presented in this study are included in the article. Further inquiries can be directed to the corresponding author.
